# Microdiscectomy Under Local Anesthesia and Spinal Block in a Pregnant Female

**DOI:** 10.7759/cureus.20241

**Published:** 2021-12-07

**Authors:** Denis Babici, Phillip M Johansen, Stu L Newman, Timothy E O'Connor, Timothy D Miller

**Affiliations:** 1 Neurology, Florida Atlantic University Charles E. Schmidt College of Medicine, Boca Raton, USA; 2 Neurological Surgery, Florida Atlantic University Charles E. Schmidt College of Medicine, Boca Raton, USA; 3 Neurological Surgery, Boca Raton Regional Hospital, Boca Raton, USA

**Keywords:** neuroanesthesia, regional anesthesia, special interest in obstetric and transplant anesthesia, mri imaging, open lumbar microdiscectomy

## Abstract

The surgical plan and the anesthetic approach are vital in determining the proper treatment of lumbar disc herniation in pregnancy. The diagnostic tools available, as well as the anesthetic agents and methods of delivery, vary in pregnant patients due to factors such as radiation exposure and hemodynamics in the patient and fetus. The gestational age also plays an important role in determining treatment options. When possible, surgery should be avoided during the first trimester, especially during the period of organogenesis, as general anesthesia can interfere with this process. However, when focal neurological deficits are present, urgent surgical decompression may be necessary. In such cases, the selection of anesthesia must be guided by maternal indications and the nature of the surgery. Maternal safety and avoidance of fetal hypoxia and subsequent preterm labor are crucial when pregnant patients receive anesthesia. As a result, local anesthesia is often preferred when possible due to the decreased risk of systemic toxicity. Decompression surgery in pregnant females with lumbar disc herniation, using a multidisciplinary approach among the surgeon, obstetrician, and anesthesiologist, is an effective and safe procedure for both the mother and the fetus. We present the case of a pregnant female at four weeks of gestation who presented with lower back pain radiating down her right leg. MRI of the lumbar spine showed large L4-5 disc herniation. She underwent a successful right L4-5 microdiscectomy under local anesthesia and spinal block using bupivacaine and was completely awake throughout the procedure. Postoperatively, she experienced immediate improvement of symptoms.

## Introduction

Low back pain is a common symptom in pregnancy and may be present in up to 56% of pregnant women [1]. Previous studies have correlated low back pain and pelvic pain with increased levels of relaxin, a hormone produced mainly by the corpus luteum during pregnancy. However, biomechanical changes, weight gain, and sagittal imbalance are also possible etiologies [2]. Radiculopathy caused by disc herniation during pregnancy is rare, affecting 1 in 10,000 pregnant women [3]. There are important considerations that need to be addressed in pregnant patients with disc herniation, such as which drugs and diagnostic tests are safe during pregnancy, the surgical indications, the ideal time and technique for the procedure, the appropriate anesthesia, and the continuation of the pregnancy after surgery. Few studies exist in the current literature regarding this subject, and at this time, widely recognized treatment protocols have not been established [4].

## Case presentation

**Figure 1:**
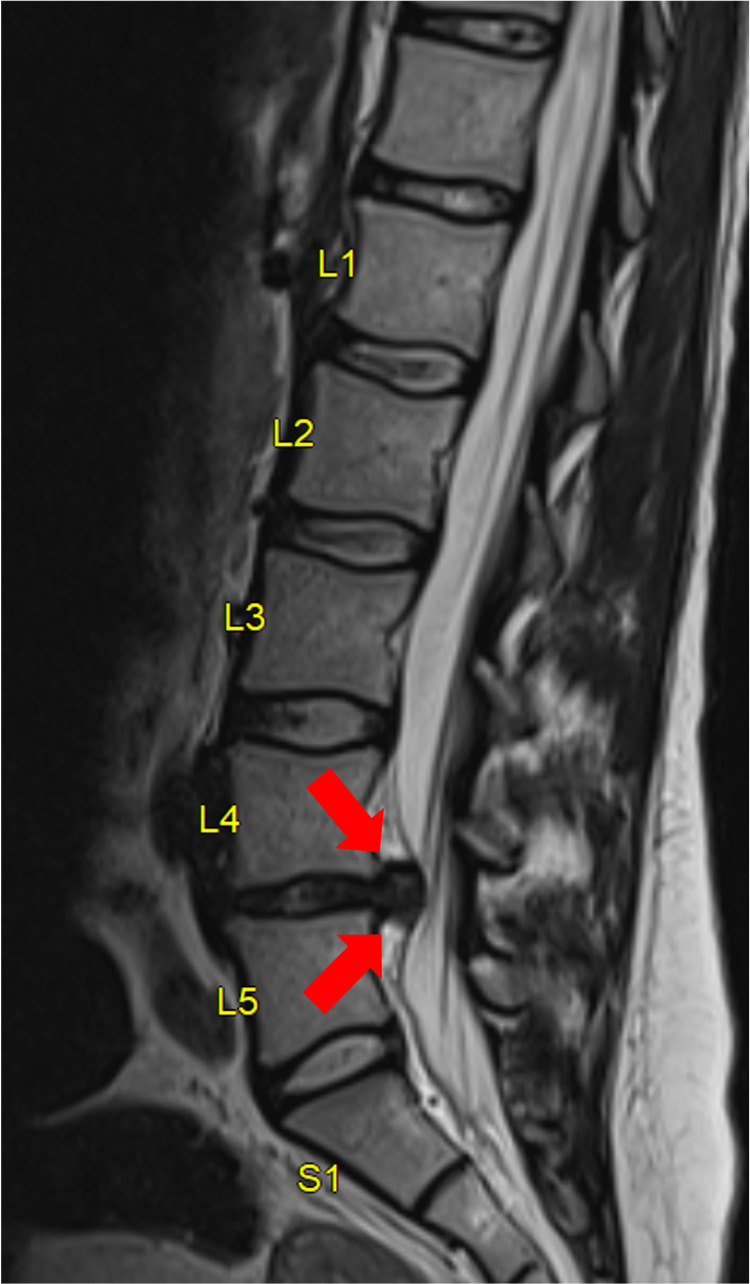
Sagittal MRI of the lumbar spine. Large paracentral L4-5 disc herniation (red arrow).

**Figure 2:**
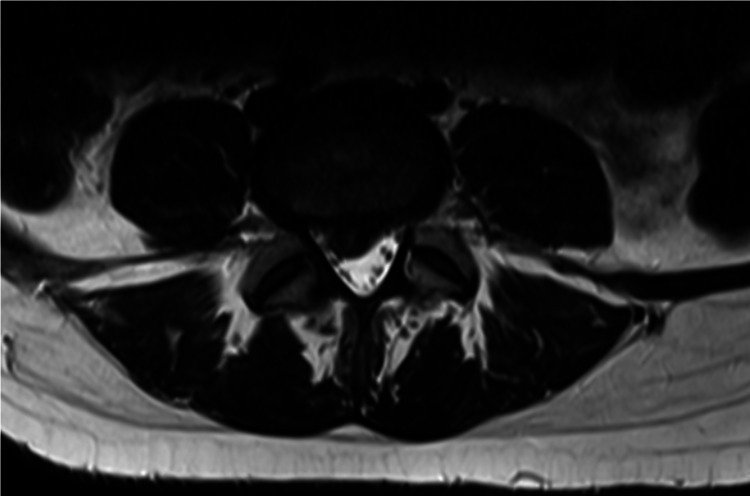
Axial MRI of the lumbar spine. Central disc extrusion extending to the right lateral recess and causing compression of the descending nerve roots.

The patient is a 32-year-old pregnant female at four weeks of gestation with no significant medical history. She presented with a four-month history of lower back pain radiating down her right leg. The patient described an aching, stabbing, and throbbing back pain severe enough to hinder ambulation, for which she presented to the emergency department. She also noted numbness and tingling in the right L5 distribution. She denied any urinary or bowel incontinence or saddle anesthesia. MRI of the lumbar spine showed large right paracentral L4-5 disc herniation with compression of the descending left L5 nerve root (Figures 1, 2).

Initially, conservative treatment was recommended, including physical therapy and pain management. However, symptoms failed to resolve despite conservative management, and the patient remained debilitated with pain and had progressive neurological deficits on examination. After discussion with Ob/Gyn and Anesthesiology, the decision was made to proceed with L4-5 microdiscectomy under spinal blockade and local anesthetic.

Description of the procedure

After informed consent was obtained, the patient was taken to the operating room. Under ultrasound guidance, using Whitacre 3.5 cm needle epidural regional block at L3-4 with 150 mg of bupivacaine was performed by the anesthesia team. The patient was completely awake and not sedated. She was transferred to the prone position and onto the Wilson frame, where a single intraoperative fluoroscopic image was used to confirm and localize the operative level. A midline incision was made, and subperiosteal dissection was used to expose the right L4 lamina. After the MLD retractor was placed at the L4-5 disc space, the second and final fluoroscopic image was obtained to confirm the level of interest. A right L4 hemilaminectomy was performed. A nerve root retractor was used to reflect the dura medially, and a 15 blade was used to create an incision in the posterior longitudinal ligament constraining the disc herniation. An extremely large, extruded disc fragment was removed with significant decompression of the thecal sac and the traversing nerve root. A Woodson was subsequently used to ensure all neural elements remained free from compression. Meticulous hemostasis was obtained, and the wound was closed in layers in standard fashion. The patient was transferred back to a supine position on the recovery bed and transported to recovery in stable condition. The duration of the procedure was 67 minutes, and the patient was completely awake and hemodynamically stable throughout the entire procedure. The procedure went well without complications, and the patient was discharged on postoperative day four with no pain in her right lower extremity.

## Discussion

There are established, well-defined guidelines for the treatment of lumbar disc herniation in non-pregnant patients. However, there are several special considerations when treating a pregnant patient with a non-obstetric disease, especially when a surgical procedure is indicated. In a non-pregnant patient with signs of radicular compression, imaging must be performed to confirm disc herniation [5]. MRI is considered the gold standard for such soft tissue pathologies. Although there are no studies demonstrating the harmful effects of MRI during pregnancy, the American College of Radiology recommends avoiding MRI during the first trimester and refraining from any associated contrast agents [6]. When MRI is necessary during the first trimester, the risks and benefits should be clearly explained to the expectant mother so she can make an informed decision [7].

The absolute indications of discectomy during pregnancy are the same as for any patient and include cauda equina syndrome or progressive neurologic deficit. Failure to perform the surgical procedure in such instances may result in permanent deficits [8]. The procedure is considered emergent in cauda equina syndrome and urgent with progressive neurologic deficits. Elective surgery in patients without neurological deficit or debilitating symptoms should be avoided during pregnancy, especially during the first trimester when organogenesis is underway. The second trimester is a more optimal time to perform surgery because the risk of complications, such as preterm labor, is the lowest during this period. The management and timing of most acute surgical procedures should mimic that for non-pregnant patients [9]. In addition, the specific anesthetic agent and method of delivery must be guided by maternal indications and the nature of the surgery. No study has found an association between improved fetal outcomes and any specific anesthetic technique, except for a single retrospective chart analysis in which the use of general anesthesia was associated with a significantly lower birth weight despite similar gestational age at delivery [9].

Pregnancy has unique risks during general anesthesia, both to the mother and the fetus [10]. For these reasons, many anesthesiologists try to avoid general anesthesia if possible and instead opt for regional anesthetics, which carry the decreased risk of systemic toxicity [4]. Maternal safety and avoidance of fetal hypoxia and subsequent preterm labor are crucial when pregnant patients receive anesthesia [11]. Pregnancy is associated with the rapid spread of spinal and epidural anesthesia. There is limited data available regarding the relative potency of a motor block with neuraxial anesthetics in non-pregnant versus pregnant women. One study showed that bupivacaine was 1.14 times more potent in pregnant versus non-pregnant women, thereby requiring more careful dosing and monitoring [12]. Despite the added precautions required when administering neuraxial anesthesia to pregnant patients, much of the existing literature surrounding neuraxial anesthesia describes experiences in obstetrics as these patients frequently insist on neuraxial blocks. Neuraxial anesthesia may also be administered after neurologic injury. Treatment during the acute phase of neurologic injuries typically requires general anesthesia to prevent further hemodynamic and respiratory deterioration. After the acute phase, neuraxial blocks may be recommended because the patient’s immediate stability is less of a factor [13].

Understanding physiological changes during the different periods of pregnancy are imperative to providing safe anesthesia. An increase in cardiac output and oxygen consumption, as well as reduced functional reserve capacity, put pregnant women at risk for desaturation and hypoxia during induction of anesthesia. The current hypothesis dictates that nearly all of the common drugs used in anesthesia can be safely used after the period of organogenesis, but detailed drug safety studies during pregnancy are lacking [14]. Regional anesthesia decreases the risk of failure to intubate and aspiration, in addition to reducing the exposure of fetuses to potentially teratogenic drugs. However, previous studies have failed to show either anesthetic technique to be superior regarding fetal outcomes [14]. Numerous studies have shown that most drugs can have teratogenic properties at certain doses and at certain times of pregnancy [15]. Studies of transplacental drug delivery in humans are difficult to perform for ethical reasons, and the applicability of animal studies as models is limited because the anatomy and function of the placenta are species-specific. Consequently, even though no anesthetic drugs are blatantly teratogenic, there is no clear anesthetic of choice during pregnancy [15,16].

## Conclusions

There are many special considerations when treating a pregnant patient with a non-obstetric disease, especially when a surgical procedure is indicated. Decompression surgery in pregnant females with lumbar disc herniation, when adhering to a multidisciplinary approach, can be an effective and safe procedure for both the mother and the fetus. The diagnosis of spinal motion segment pathology, and its surgical treatment, is characterized by several features and, in some cases, cannot be cured during pregnancy. However, when focal neurological deficits or debilitating symptoms are present, awake decompression surgery can be performed in a safe and effective manner.
